# Women Ornament Themselves for Intrasexual Competition near Ovulation, but for Intersexual Attraction in Luteal Phase

**DOI:** 10.1371/journal.pone.0106407

**Published:** 2014-09-02

**Authors:** Jin-Ying Zhuang, Jia-Xi Wang

**Affiliations:** Key Laboratory of Brain Functional Genomics (MOE & STCSM), School of Psychology and Cognitive Science, East China Normal University, Shanghai, China; Knox College, United States of America

## Abstract

The present study examined women's attentional bias toward ornamental objects in relation to their menstrual phase as well as to motivations of intersexual courtship or intrasexual competition. In Experiment 1, 33 healthy heterosexual women were tested in a bias-assessment visual cuing task twice: once on a high-fertility day (during the ovulatory phase) and once on a low-fertility day (during the luteal phase). They paid greater attention to pictures of ornamental objects than to pictures of non-ornamental objects near ovulation, but not during the luteal phase, suggesting an ornamental bias during the high-fertility phase. In Experiment 2, before the visual cuing task, 40 participants viewed 10 same-sex or opposite-sex facial photographs with either high or low attractiveness as priming tasks to activate the intrasexual competition or intersexual courtship motives. Results showed that women's ornamental bias was dependent on the interaction of menstrual phase and mating motive. Specifically, the ornamental bias was observed on the high-fertility day when the subjects were primed with high-attractive same-sex images (intrasexual competition) and was observed on the low-fertility day when they were primed with high-attractive opposite-sex photographs (intersexual courtship). In conclusion, the present findings confirm the hypothesis that, during the high-fertility phase, women have an attentional bias toward ornamental objects and further support the hypothesis that the ornamental bias is driven by intrasexual competition motivation near ovulation, but driven by intersexual courtship motivation during the luteal phase.

## Introduction

Women seek beautification through ornamentation. Compared to men, women spend considerably more time and money on a very particular class of products, namely clothes, jewelry, and other fashion accessories, to enhance their physical appearance [1]–[3]. For example, in the USA alone, women spend well over $100 billion annually on fashion apparel [4], and in 2008, the amount spent was 50% more than the entire US government spent on education [5].

Augmenting one's attractiveness through ornamentation may provide general advantages for women in social activities. However, several recent studies have related these ornamental behaviors to their biological, presumably reproductive, roots. For example, women were judged as more likely to appear to be “trying to look more attractive” in photographs taken during their fertile phase (near ovulation) than in those taken during the non-fertile (luteal) phase [6]; preferred more revealing and sexier clothing, and were more likely to choose appearance-enhancing products (i.e., sexy clothes and accessories) during the fertile phase compared to during the luteal phase [5], [7]. Similarly, Saad and Stenstrom [8] found that while women's food-related desires, dollars spent, and eating behaviors were greater during the luteal phase, appearance-related desires, dollars spent, and beautification behaviors increased near ovulation. Grammer et al. [9] found that estrogen (fertility) levels correlated positively with the amount of skin revealed and clothing tightness of woman patrons at an Austrian night club.

In sum, the fertility phase of women appears to have a positive relationship with appearance-related styling behaviors. However, in considering the putative association between hormonal levels and women's ornamental behaviors, there are numerous external factors and constraints that must be contemplated, such as differences in individual style preferences, the availability of goods, and the context of an experimental environment, etc.

The first aim of this study was to explore the relationship between the fertility phase and women's ornamental behaviors more rigorously by testing the effect of two menstrual phases (ovulatory *vs*. luteal) on the lower order cognitive process of attention. Ecological theories of social cognition suggest that lower order cognitive processes [10], such as attention, are tuned adaptively to processing key features in the environment that are relevant to the satisfaction of currently important motives [11]. In turn, motivational states can promote attentional biases such that perceivers are relatively inefficient at disengaging their attention from goal-relevant stimuli. For example, using a visual cuing task, Miller and Maner [12] asked subjects to categorize objects as a circle or a square as soon as possible, with each category object following a target picture of a disfigured or normal face either in the same location as the face (filler trials) or a different quadrant (attentional-shift trials). They found that participants who had been ill recently were slower to shift their attention away from the location of disfigured faces than were non-recently ill participants. The heightened attention to disease cues in the recently ill group, as evidenced by longer attentional shift latencies, indicated a motivation of protection from contagion. Similarly, Maner et al. [13] found that the activation of mate-search or mate-guarding motive led subjects to perseverate on physically attractive members of the opposite sex (potential mates) or one's own sex (potential rivals). Thus, if changing hormone levels in women lead to fluctuations in ornamental motives, their attention to ornamental stimuli in the environment should change accordingly.

A similar visual cuing task was employed in this study to examine the change of ornamental motives around women's menstrual cycle. According to the operational definition of human ornamentation [6], as accepted herein, we included products such as cosmetics, jewelry, and fashionable clothes as ornamental stimuli [5]–[7]. We hypothesized that if women had stronger motivation for ornamentation near ovulation than during the luteal phase, they would have exhibited longer attentional shift latencies from ornamental objects than from non-ornamental objects.

Additionally, self-beautification through ornamentation near ovulation has been suggested to being related to mate attraction [5]–[7]. Several competing hypotheses have been proposed mediating the relationship between women's ornamentation and mate attraction. The first one is the intersexual courtship hypothesis, which posits that because men value youth and attractiveness for reproduction, women should advertise those qualities to attract high quality mates [14]. A handful studies support this notion. For example, building upon the observation that mimicry increased women's attractiveness to men, Guéguen found that women near ovulation mimicked men better than during the luteal or menstrual phase [15], [16]. The amount of time women spent walking in front of a male associate was longer and their gait was rated as subjectively sexier when near ovulation, compared with the non-fertile phase [17]. Women during their fertile phase have been found to have a larger mean pupil diameter when viewing sexually significant stimuli than when in a non-fertile phase [18] and highly dilated pupils in women have been rated as “more feminine”, “prettier”, and “softer” by men [19]. Additionally, men in gentlemen's clubs gave more tips to professional lap dancers during their fertile phase, possibly due to the increased attractiveness by nonverbal behaviors displayed by women [20].

Fisher [21] proposed an alternate motivation for ornamentation behavior near ovulation in women, namely intrasexual competition. According to sexual selection studies, the competition strategy used by human females is primarily one of self-promotion involving epigamic displays of physical attractiveness, such as wearing make-up or sexy clothing, and derogation of the appearance of their potential rivals [22]–[27]. Interestingly, when Fisher [21] asked women to rate other women's facial attractiveness, she found that the ratings were at their nadir near ovulation, suggesting that they were derogating the competitors when the reproductive stakes were highest. Similarly, Durante et al. [5] found that women's preference for sexy products during the high fertility phase was greater in women primed of intrasexual competition with photographs of local attractive women.

In this study, our second aim was to explore the specific motives affecting the ornamental biases across the menstrual cycle. We used the established priming methodology wherein women were shown photographs of highly attractive men or women to activate intersexual courtship or intrasexual competition motivations subliminally [5], [28]–[31]. Though competition and courtship goals can be activated most strongly when potential mates or same-sex competitors are rated as highly attractive [29], considering that the effect of fertility was suppressed when women were primed with unattractive women or with men of high or low attractiveness in the research of Durante et al. [5], we also included photographs with low attractiveness in the priming conditions to get a sufficient understanding of women's ornamental biases across the menstrual cycle.

In Experiment 1, we tested the hypothesis that, when in the high-fertility phase (near ovulation), women paid greater attention to pictures of ornamental objects than to pictures of non-ornamental objects. In Experiment 2, we probed how such attentional bias toward ornamental pictures was influenced by the interaction of menstrual phase (ovulatory *vs*. luteal) and motivation of the intrasexual competition or the intersexual courtship.

## Experiment 1

### Materials and methods

#### Participants

The study cohort included 33 healthy heterosexual female undergraduates at a local university who were 19 to 25 years of age (mean age  = 21.73 years, standard deviation [SD]  = 1.48). Before the experiment, the participants were surveyed with a questionnaire about their menstrual cycle regularity and cycle length within last 3 months, the date of the last menstrual onset, sexual orientation, and the use of any form of hormones including oral contraceptives and other drugs to regulate the cycle. Only participants who were heterosexual, reported that they had 28–30-day menstrual cycle, and affirmed that they were not using any form of hormones including hormonal contraceptives within last 3 months were included in this study. After the initial screening, participants were accidently asked to come to the laboratory to conduct another experiment on two separate occasions (near ovulation and during the luteal phase) with a cover story. We used the backward counting method to predict the day of ovulation and luteal phase, since this method has been successful in predicting other effects of theoretical interest [32]–[36] and generally regarded as a more accurate method of estimating cycle positions and fertility status [37]–[39]. The ovulatory phase included days between 14 and 16 days prior to a woman's next predicted menstrual onset, and the luteal phase included days between 6 and 8 days prior to a woman's next predicted menstrual onset (next menstrual onset was predicted based on the cycle information women provided at the survey). If a woman's next predicted menstrual onset was between 8 and 14 days away, she was scheduled to complete the luteal session first (*n* = 15); if not, she was scheduled to complete the ovulatory session first (*n* = 18). Written informed consent was obtained from all participants and the protocol used in the current study was reviewed and approved by Ethics Committee of East China Normal University.

#### Stimuli

**Figure 1:**
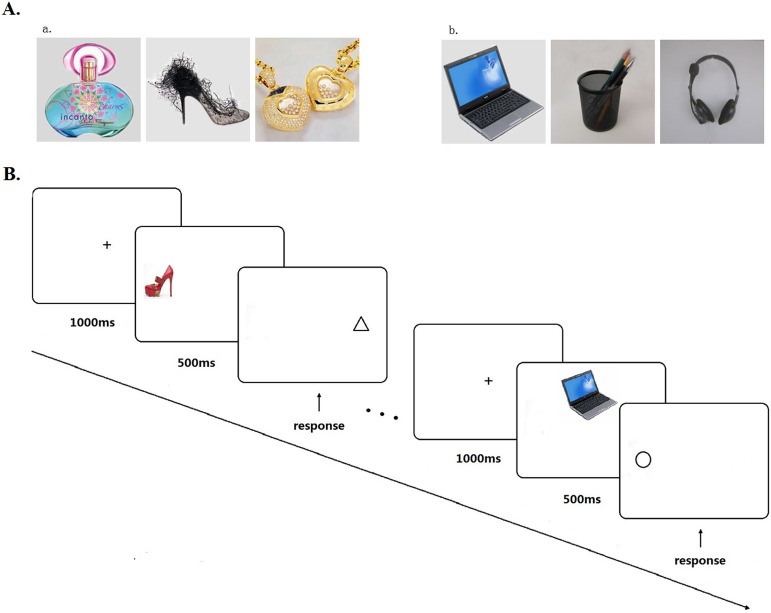
Visual cuing task paradigm. (A) a. Examples of ornamental objects; b. examples of non-ornamental objects. (B) Summary of task procedure.

The target stimuli consisted of 32 color pictures of ornamental objects (Fig. 1A: a) and 32 color pictures of non-ornamental objects (Fig. 1A: b). The ornamental objects include items that women adorn themselves with in their daily lives to increase their attractiveness, such as a bottle of perfume, a pair of beautiful shoes, a silver necklace, etc. The non-ornamental objects include affectively neutral every-day items that are matched, on average, on color, novelty, and volume/size, etc. with the pictured ornamental objects, but not used to enhance personal attractiveness, such as a drinking glass, a laptop computer, etc. All pictures were collected from websites or photographed by experimenters. In each picture, the objects were centrally positioned against a constant gray background. All images were cropped to a size of 200×200 pixels and the lightness of each image was adjusted to the same standard by using Adobe Photoshop software.

Another 21 participants (5 male, 16 female; mean age  = 22.9, SD = 1.44; ranging from 21 to 26 years old) who were blind to the experiment rated all objects in the pictures (64 in total) on values of attractiveness, novelty and utility, indicating to what extent they thought the objects were on a 7–point Likert scale (ranging from 1 =  the lowest value to 7 =  the highest value). The participants were also asked to judge whether the objects were ornamental for women or not. All subjects agreed on that the 32 ornamental objects were ornamental for women and the others were not. For the rating scores on attractiveness, utility and novelty, paired samples *t* tests showed that ornamental objects were rated as more attractive than non-ornamental objects (*t* = 4.32, *p*<.001; ornamental: M = 4.32, SD = 1.37; non-ornamental: M = 2.56, SD = 1.26), but were similar in utility (*t* = −.87, *p* = .39; ornamental objects: M = 3.73, SD = 1.50; non-ornamental objects: M = 4.12, SD = 1.44) and novelty (*t* = .74, *p* = .46; ornamental objects: M = 2.67, SD = 1.12; non-ornamental objects: M = 2.41, SD = 1.18).

#### Visual cuing task

Participants completed the visual cuing task [12], [13] on a laptop computer (14-in) with a refresh rate of 60 Hz, working individually. The distance between the monitor and the eyes of subjects was 45 cm. At the beginning of each trial, a fixation cross (0.5 cm×0.5 cm) appeared on the center of the computer screen for 1,000 ms. Next, a target stimulus image was displayed for 500 ms in one quadrant of the screen. As soon as the target picture disappeared, a categorization object (circle or triangle, 1.5 cm×1.5 cm) appeared immediately in either the same location as the picture (filler trials) or in a different quadrant (attentional-shift trials). When the categorization object appeared, the participants had to indicate as quickly and accurately as possible whether the object was a circle or a triangle by pressing the *A* or *K* key, respectively, on the keyboard (see Fig. 1B). Longer latencies on attentional-shift trials indicated a more extended time for shifting attention away from the location where the target picture had been. After completing 20 practice trials, participants completed 192 experimental trials (33.3% filler, the ratio used by Maner et al., 2007). Each stimulus item was displayed to each subject three times. The order of trials and the categorization objects (circle or triangle) were randomized.

### Results

#### The effect of session order

Trials with incorrect categorization responses (1.51% of the trials) and trials with latencies that were more than three SDs above or below a participant's mean latency (1.25% of all trials) were excluded.

Previous researchers have indicated that hormonal condition at first exposure to stimuli may affect subsequent interest in the stimuli over the other hormonal conditions [40], [41]. The effect of session order (the order of first entry into the test) was examined first. A 2 (menstrual phase: ovulatory *vs.* luteal) ×2 (target type: ornamental object *vs.* non-ornamental object) ×2 (initial phase: ovulatory phase first *vs.* luteal phase first) mixed ANOVA was performed on mean response latencies for categorization of objects on attentional shift trials. Any effect with the session order was not significant, therefore, the factor of session order was dropped from further analyses.

#### Effects of target type and menstrual phase on attentional bias

**Figure 2:**
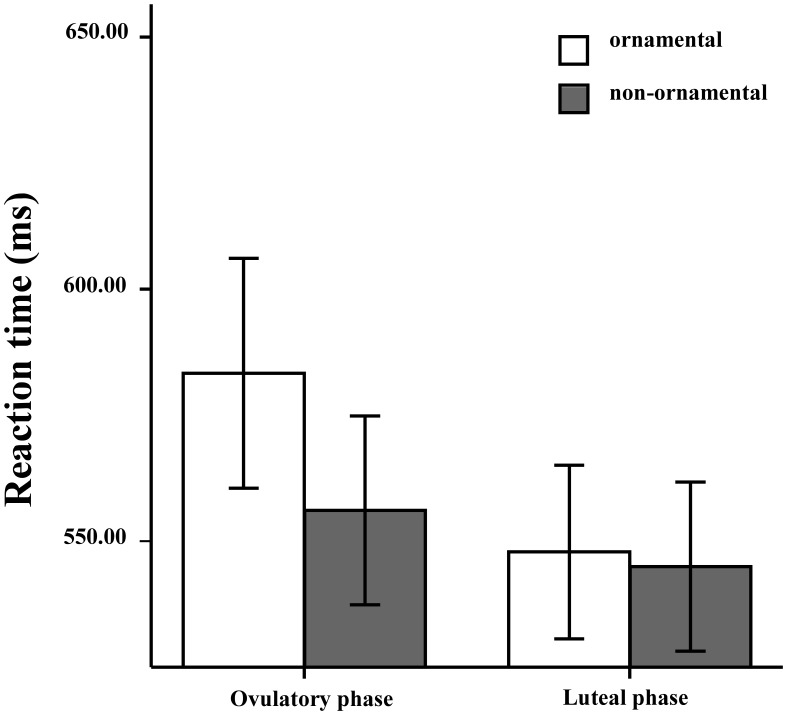
Experiment 1. Mean reaction time for categorization of objects in attentional-shift trials in the visual cuing task as a function of target type (ornamental *vs*. non-ornamental) and menstrual phase (ovulatory *vs*. luteal) (error bars show 1 S.E.M.).

A repeated measures ANOVA of mean response latency for categorization of objects with target type (ornamental objects *vs*. non-ornamental objects) and menstrual phase (ovulatory *vs*. luteal) as within-subjects factors in attentional-shift trials revealed a main effect of target type (*F*_1,32_ = 18.26, *p*<.001, η*_p_^2^* = .36), but not of menstrual phase (*F*_1,32_ = 1.55, *p* = .22, η*_p_^2^* = .046). The interaction was significant (*F*_1,32_ = 11.46, *p* = .002, η*_p_^2^* = .26). As shown in Figure 2, simple effects analysis showed that participants attended more to ornamental pictures (*M* = 583.31 ms, *SD* = 131.04) than to non-ornamental pictures (*M* = 556.11 ms, *SD* = 107.69) when tested during the ovulatory pahse, *F*_1, 32_ = 22.37, *p*<.001, η*_p_^2^* = .41. Conversely, participants displayed no such attentional bias during the luteal phase (ornamental: *M* = 547.84 ms, *SD* = 98.98; non-ornamental: *M* = 544.96 ms, *SD* = 96.43; *F*_1,32_ = 0.47, *p* = .50, η*_p_^2^* = .015).

#### Implications of the attractiveness of ornamental objects

Considering that the attractiveness ratings of ornamental objects in the visual cuing task was greater than that of non-ornamental objects, we evaluated whether the attentional bias to ornamental objects near ovulation was purely due to the attractive features of the objects in the task with regression analyses. First, the mean difference of response latencies on attentional-shift trials on each object between the ovulatory phase and luteal phase was calculated and then regressed onto the mean rating score of the attractiveness of that object. The effect was not significant (β = .27, *p* = .095). Second, the mean difference of the response latency was regressed onto either the attractiveness rating score of ornamental objects or non-ornamental objects separately. The effect bordered significance for the ornamental objects (β = .37, *p* = .075), but not for the non-ornamental objects (β = .12, *p* = .67), suggesting that a possible contribution of object attractiveness to the attentional bias near ovulation may only take place within the ornamental objects.

### Discussion

The results of Experiment 1 showed that women attended more to ornamental objects than to non-ornamental objects near ovulation, replicating the ornamental bias effect, but with cognitive-based evidence. This bias dissipated during the luteal phase. Since female attractiveness plays an important role in mate attraction, it is quite adaptive for reproduction-related hormones to initiate pro-ornamentation motivation in women to attract high quality mates during their most fertile stage.

However, to ascertain whether this ornamental bias was driven by intersexual courtship or intrasexual competition motivation, in the next experiment, we primed the motivation of women subjects first and then asked them to finish the same visual cuing task as in Experiment 1.

## Experiment 2

### Materials and methods

#### Participants

Forty healthy heterosexual female participants (mean age  = 22.70 years, SD = 1.95, range 19–26 years old) were recruited with the same methods and requirements as in Experiment 1. Ten participants were assigned randomly to each of four priming conditions: highly attractive female condition, low attractive female condition, highly attractive male condition, and low attractive male condition. Written informed consent was obtained from all participants. The protocol of the experiment was approved by Ethics Committee of East China Normal University.

#### Priming task

The priming stimuli were colorful front-face photographs of either 10 highly attractive or low-attractive men or women collected from a local website (http://www.renren.com/SysHome.do) or photographed by experimenters. The photographs were selected from a larger set of photos (n = 100 for male or female each) that were pre-rated on physical attractiveness (using a 9-point scale ranging from 1 =  low attractiveness to 9 =  high attractiveness) by an independent sample of 16 female students (mean age  = 20.90 years, SD = 1.32) who were blind to the purpose of this experiment and did not participate in the other parts of this experiment. The 10 highly attractive male or female photographs selected were more than two SD above the mean scores of attractiveness ratings (men: M = 4.0, SD = 1.37; women: M = 4.24, SD = 1.23, respectively) to make sure that they were really looked attractive; the attractiveness scores for the 10 males or females with low attractiveness were over 1 SD below the mean scores of the attractiveness rating. We did not set the standard to select photographs with 2 SD below the mean scores of the attractiveness rating, because so it would have been made an extremely unattractive sample.

#### Experimental design and procedures

A 2 (sex of prime: male *vs*. female, between subjects factor) ×2 (attractiveness of prime: high *vs*. low, between subjects factor) ×2 (menstrual phase: ovulatory *vs*. luteal, within subjects factor) ×2 (target type: ornamental objects *vs*. non-ornamental objects, within subjects factor) mixed factorial design was employed. Before the visual cuing task, participants viewed and rated 10 photographs in a random order, one by one, according to which priming condition they were assigned to. Participants rated the 10 individuals on attractiveness, friendliness, and extraversion having been given the cover story that we were interested in learning about several different things, including people's ability to judge attractiveness. After the priming task, participants completed the same visual cuing task as in Experiment 1. All participants performed the priming and visual cuing task twice—once near ovulation and once during the luteal phase. Applying the same randomization procedures as in Experiment 1, about half (n = 24) of the participants completed these tasks near ovulation first and about half (n = 16) completed the tests first during their luteal phase.

### Results

Trials with incorrect categorization responses (2.08% of the trials) and trials with latencies that were more than three SDs above or below each participant's mean latency (0.70% of all trials) were excluded. The mean reaction time or response latency for object categorization in attentional shift trials was calculated for each participant as a dependent variable (see Table 1).

**Table 1:** Mean reaction times for object categorization in attentional shift trials.

Priming		Ovulatory phase				Luteal phase			
Sex	Attractiveness	Ornamental		Non-ornamental		Ornamental		Non-ornamental	
		M	SD	M	SD	M	SD	M	SD
**Male**	high	587	26	580	20	571	75	539	74
	low	519	48	533	69	539	82	532	103
	total	553	51	556	55	555	78	535	87
**Female**	high	471	48	447	54	497	108	487	139
	low	524	62	515	79	555	49	540	44
	total	498	60	481	75	526	87	514	104

The effect of session order was examined first. A 2 (menstrual phase: ovulatory *vs.* luteal) ×2 (target type: ornamental object *vs.* non-ornamental object) ×2 (initial phase: ovulatory phase first *vs.* luteal phase first) mixed ANOVA was performed on the dependent variable. Any effect with the session order was not significant, therefore, the factor of session order was dropped from further analyses.

In order to investigate the effect of interest, a mixed ANOVA with the sex (male *vs*. female) and attractiveness (high *vs.* low) of the prime as between-subjects factors and menstrual phase and target type as within-subjects factors was conducted on the mean response latencies for categorization of objects on attentional shift trials. The three-way interaction (sex of prime × menstrual phase × target type) was significant (*F*_1,36_ = 6.05, *p* = .019, η*_p_^2^* = .144). Based on our interest, simple effects analyses of the three-way interaction showed that, during the ovulatory phase, when the priming stimuli were females, the participants significantly preferred ornamental objects to non-ornamental objects (*F*_1,19_ = 12.84, *p* = .002, η*_p_^2^* = .40); whereas when the priming stimuli were males, there was no such significant difference in preference (*F*_1,19_ = .29, *p* = .59, η*_p_^2^* = .015). Conversely, during the luteal phase, when the priming stimuli were males, there was a significant preference for ornamental objects over non-ornamental objects (*F*_1, 19_ = 7.81, *p* = .012, η*_p_^2^* = .29); whereas when the priming stimuli were females, there was no such significant preference (*F*_1,19_ = 3.72, *p* = .07, η*_p_^2^* = .16) (see full report at Table S1 in File S1).

**Figure 3:**
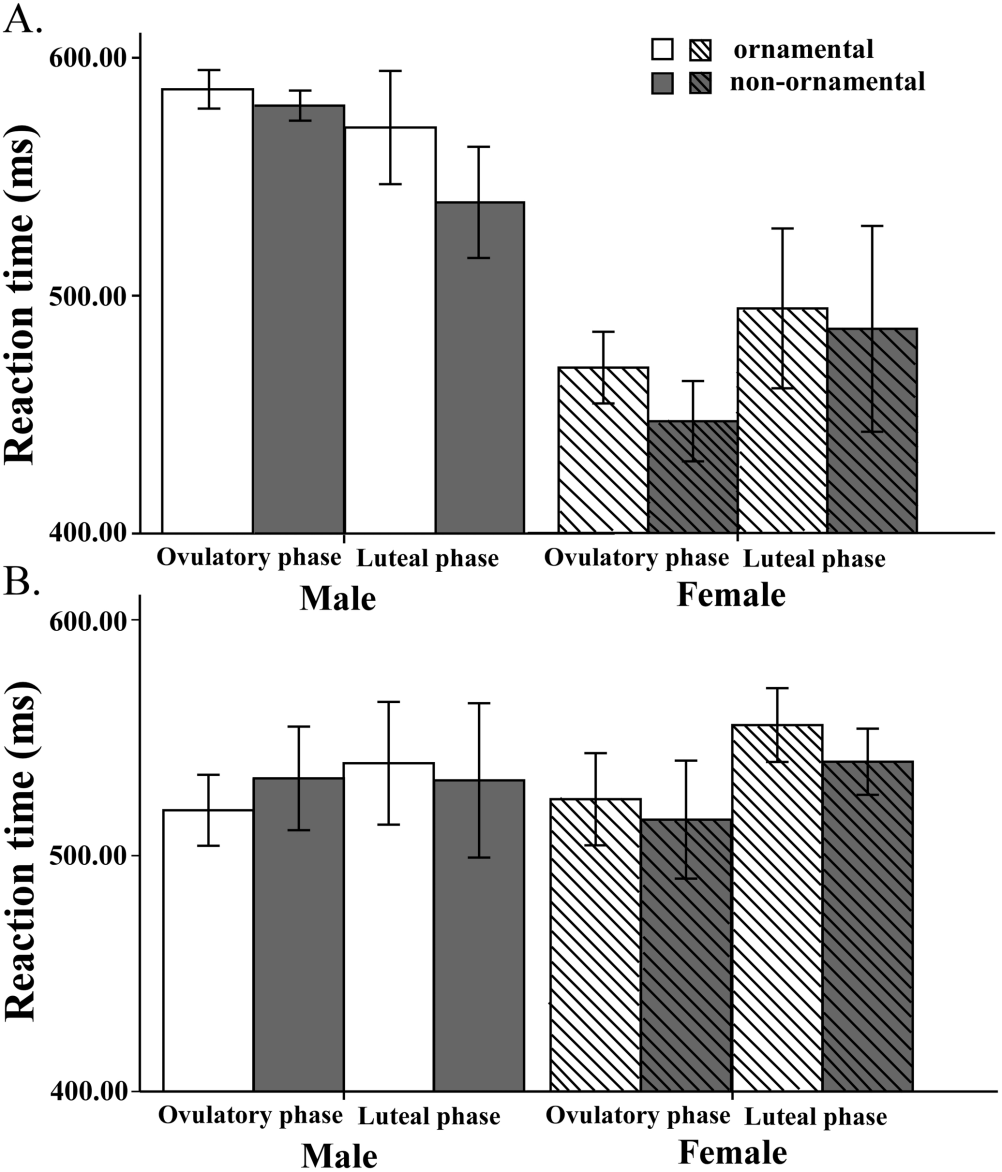
Experiment 2. Mean reaction time for categorization of objects in attentional-shift trials in the visual cuing task as a function of target type (ornamental *vs*. non-ornamental) and menstrual phase (ovulatory *vs*. luteal) in the highly attractive (A) and low-attractive (B) priming conditions (error bars show 1 S.E.M.).

The two-way interaction was observed on target type × attractiveness of prime (*F*_1,36_ = 4.51, *p* = .041, η*_p_^2^* = .11) and on sex of prime × attractiveness of prime (*F*_1,36_ = 5.81, *p* = .021, η*_p_^2^* = .14). Simple effects analyses of the interactions indicated that when the priming stimuli were highly attractive photographs, the response latencies were longer for ornamental objects (*F*_1,39_ = 23.02, *p*<.001, η*_p_^2^* = .37) than for non-ornamental objects, as well as for male primes (*F*_1,78_ = 29.38, *p*<.001, η*_p_^2^* = .27) than for female primes (see Figure 3A); however, when the priming stimuli were low-attractive photographs, there was no such significant difference between ornamental and non-ornamental objects (*F*_1,39_ = .85, *p* = .36, η*_p_^2^* = .021), as well as between the male and female primes (*F*_1,78_ = .034, *p* = .85, η*_p_^2^* = .00) (see Figure 3B).

The sex of prime (*F*_1,36_ = 5.15, *p* = .029, η*_p_^2^* = .125) and target type (*F*_1,36_ = 12.53, *p* = .001, η*_p_^2^* = .26) were confirmed to be significant main effects. All the other effects were not significant.

Based on our results of the two-way interaction and previous claims that the motivation of intrasexual competition or intersexual courtship can be activated most strongly when potential mates or same-sex competitors are rated as highly attractive [29], a further mixed ANOVA with the sex of prime, the menstrual phase and target type as independent factors within the highly attractive priming conditions confirmed the significant effect of three-way interaction (sex of prime × menstrual phase × target type: *F*_1,18_ = 11.44, *p* = .003, η*_p_^2^* = .39). Simple effects analysis showed that during the ovulatory phase, when the priming stimuli were highly attractive females (intrasexual competition), participants significantly preferred ornamental objects to non-ornamental objects (*F*_1,9_ = 27.11, *p* = .001, η*_p_^2^* = .75), whereas when the priming stimuli were highly attractive males (intersexual courtship), no such preference was found (*F*_1,9_ = 1.89, *p* = .20, η*_p_^2^* = .17). Conversely, during the luteal phase, when the priming stimuli were highly attractive males, participants significantly preferred ornamental objects to non-ornamental objects (*F*_1,9_ = 36.17, *p*<.001, η*_p_^2^* = .80), whereas when the priming stimuli were highly attractive females, no such preference was found (*F*_1, 9_ = .71, *p* = .42, η*_p_^2^* = .07) (see Figure 3A; a detailed report in Table S2 in File S1). Though the pattern was the same, the robustness of the effect was increased when the analysis was conducted within the highly attractive priming conditions. For the conditions with low-attractiveness primes, the same analysis yielded no significant effects (see Figure 3B; a detailed report in Table S3 in File S1).

### Discussion

The results of Experiment 2 showed that the attentional bias towards ornamental objects in women was dependent on the interaction of menstrual phase and priming conditions. Specifically, an attentional bias for ornamental objects was observed on subjects' high-fertility day (near ovulation) only when they were primed of intrasexual competition motivation. Whereas, the attentional bias for ornamental objects was observed on subjects' low-fertility day (during luteal phase) only when they were primed of intersexual courtship motivation. Any effect was not observed with the prime of low-attractive photos. These results suggested that the ornamental bias was motivated by an intrasexual competition strategy near ovulation, but was motivated by an intersexual courtship strategy during the luteal phase.

## General Discussion

In a visual cuing task, when female subjects were presented randomly with ornamental and non-ornamental pictures, they attended more to ornamental objects than to non-ornamental objects during their high-fertility phase. According to ecological theories about the relationship between attention and motivation [10], [11], the attentional bias towards ornamental objects reflects participants' heightened motivation for attractiveness enhancement through ornamentation near ovulation. Since female attractiveness plays an important role in mate attraction, it is quite adaptive for reproductive-related hormones to initiate pro-ornamentation motivation in women during their peak fertility phase. Our results confirmed the ornamentation bias across menstrual cycle from previous research and provided further evidence on the cognitive level.

Using highly attractive male or female photographs as priming stimuli to implicitly activate the intersexual courtship or intrasexual competition motivation, the results of Experiment 2 showed that the ornamental bias during women's high-fertility phase was observed only under the intrasexual competition condition, providing evidence for the previous claim that women's ornamentation near ovulation acts as a proverbial “deer's antlers”, mediating a same-sex competition function.

To be specific, though the ultimate goal of women's ornamentation near ovulation is to attract high-quality mates to bestow genetic benefits upon one's offspring, one's relative attractiveness within a social group may be more important than her actual individual traits. A relatively more attractive female in a group should render herself more desirable to members of the opposite sex relative to others of the same sex with the same goal [42]. Evidence indicates that women are indeed sensitive to the physical attractiveness of same-sex competitors. For example, women perceived other attractive women to be greater threats to their relationships [43], judged a rival with high facial or bodily attractiveness to be highly distressing [44], downgraded themselves as less desirable partners after viewing facial photographs of attractive women [45], and used vocal femininity to track the threat potential of competitors since the acoustic parameters of a feminine voice have a strong effect on men's preferences [46].

Our finding that the ornamental bias was not observed under conditions of intersexual courtship during the high-fertility phase may seem somewhat counter-intuitive since the purpose of ornamentation has been thought to be mate attraction. Evidence from other animal species indicates that visually conspicuous signals or ornaments expressed by females can evolve independently of mate selection [47]. Competition for resources such as food, nest sites, water, or helpers [48]–[50] will also be linked to visually conspicuous signals in females, suggesting that these signals may function primarily in communication with other females [51]–[54]. For example, although a function of reproductive advertisement is commonly assumed for female scent marking, odor communication between females has been suggested to reduce competition for preferred males by facilitating avoidance of estrus synchrony in ring-tailed lemurs [55]. Therefore, women's ornamentation during their high-fertility phase may function primarily in competition with other women for high-quality men, rich food, or other resources. However, we must be very careful in making any direct behavioral comparisons across highly divergent species. More research is needed to probe the intrasexual competition mediated ornamental biases of human females during their high fertile phase.

Interestingly, a strong ornamental bias was observed first in this study during women's luteal phase when primed with intersexual courtship motivation. The change of the motivation from same-sex competition to opposite-sex attraction during the low-fertility phase suggests that when conception is less possible, women may act to attract sexual interest of the opposite-sex, a strategy similar to concealed ovulation and extended sexuality (women's sexual receptivity outside of the fertile window). Several studies have suggested that, during the non-fertile phase, females will use strategies such as concealed ovulation or extended sexuality to obtain broader benefits from males [56], strengthen pair bonding, or secure biparental care [57]. Men typically possess imperfect knowledge of the fertility status of women. When females lack extended sexuality, males have little incentive to remain sexually interested beyond estrus. By contrast, when females are sexually proceptive even when they are not fertile, they can maintain the sexual interest of their partners and obtain the benefit through partner-delivered direct care, food, sexual protection [58], and enhanced paternity assurance [59]. Results from a study of 50 heterosexual couples showed that women were more likely to initiate sex in their luteal phase, when they perceived their partners' investment to be lagging behind their own, than in the fertile phase [60].

Our finding that women had increased motivation for ornamentation during their luteal phase when primed with intersexual courtship cues may reflect a strategy similar to concealed ovulation or extended sexuality for two reasons. First, women are generally perceived as being less attractive during the luteal phase than during the ovulatory phase. For example, women's faces were judged by others to be less attractive [61], and the skin color less lightening [62] during the luteal phase than during the ovulatory phase. Soft tissue structures, such as the breasts, fingers, and ears, became less symmetrical [63], and women's body scents [64], [65] and voices [66] were rated as least attractive during the luteal phase. Thus, in order to maintain high-quality mates' investment in them without reproductive benefits, women may take effort to sustain their physical attractiveness through strategies such as ornamentation during their non-fertile periods. This hypothesis needs to be tested directly in the future.

Secondly, men may possess adaptive abilities to recognize subtle cues of a woman's fertility with behavioral consequences. Consistent with this perspective, a handful studies have revealed that men tend to rate certain characteristics of women (e.g., their scent, their voice, their face) as most attractive during periods of peak fertility [61],[66],[67]. In addidion, Gangestad et al. [68] and Haselton and Gangestad [33] found that women reported heightened mate-guarding behaviors by their male romantic partners during periods of peak fertility. Thus, during the luteal phase, women may feel a lag in investment from their partners and therefore may act to increase their attractiveness through ornamentation in order to maintain their mates' attraction towards them.

This research has limitations worth noting. Firstly, we used the reverse cycle day method to predict the day of ovulation and the day of luteal phase. Although this method has been successful in predicting other effects of theoretical interests [32]–[36] and we used a very narrow window (3 days) to predict the cycle positions, determining hormone levels with a more stringent method, such as the analysis of salivary samples, may help to augment the accuracy of our prediction on menstrual phases as well as to avoid the “cherry picking” effect [38], [39]. Second, the ornamental and non-ornamental pictures may not have been perfectly matched, especially on the dimension of visual attractiveness. However, our two-phased repeated measures, with phase-specific effects, should overcome any confounding effects due to stimulus mismatching on any of these descriptive dimensions. In addition, the possible influence of objects' visual attractiveness on the attentional bias was restricted to one category (the ornamental objects) excluded the possibility that any dimension other than the ornamental-non-ornamental dimension can become the main features influencing the attentional bias across the menstrual cycle.

In conclusion, the present findings confirmed the hypothesis that during the high-fertility phase, women have an attentional bias toward ornamental objects as well as the hypothesis that the attentional bias toward ornamental objects is driven by women's intrasexual competition near ovulation and is driven by intersexual courtship motivation during the luteal phase.

## Supporting Information

File S1 **Tables S1, S2, and S3.**
(DOCX)
