# Supplementary material for: Women Ornament Themselves for Intrasexual Competition near Ovulation, but for Intersexual Attraction in Luteal Phase
Source: PLoS One. 2014 Sep 2;9(9):e106407. doi: 10.1371/journal.pone.0106407 (PMC4152269; doi:10.1371/journal.pone.0106407)
Supplement: File S1 — Tables S1, S2, and S3. (DOCX) [file pone.0106407.s001.docx]

**Table S1. Results of a mixed ANOVA for the design of Experiment 2.**

| Source | *df* | F | Sig | η*_p_^2^* |
| --- | --- | --- | --- | --- |
| MP | 1, 36 | .84 | .37 | .023 |
| TT | 1, 36 | 12.53 | .001** | .258 |
| SP | 1, 36 | 5.15 | .029* | .125 |
| AP | 1, 36 | .24 | .63 | .007 |
| MP × SP | 1, 36 | 3.02 | .09 | .077 |
| MP × AP | 1, 36 | .52 | .47 | .014 |
| TT × SP | 1, 36 | 1.02 | .32 | .027 |
| TT × AP | 1, 36 | 4.51 | .04* | .111 |
| MP × TT | 1, 36 | 3.01 | .09 | .077 |
| SP × AP | 1, 36 | 5.8 | .02* | .14 |
| MP × SP × AP | 1, 36 | .87 | .36 | .024 |
| TT × SP × AP | 1, 36 | 1.95 | .17 | .051 |
| MP × TT × SP | 1, 36 | 6.05 | .019* | .144 |
| MP × TT × AP | 1, 36 | .68 | .42 | .018 |
| MP × TT × SP × AP | 1, 36 | 1.38 | .25 | .037 |

MP = menstrual phase; TT = target type; SP = sex of prime; AP = attractiveness of prime

**Table S2. Results of a mixed ANOVA with high attractive primes in Experiment 2.**

| Source | df | F | Sig. | η*_p_^2^* |
| --- | --- | --- | --- | --- |
| MP | 1, 18 | .018 | .89 | .001 |
| TT | 1, 18 | 20.05 | ∠.001** | .527 |
| SP | 1, 18 | 9.52 | .006** | .346 |
| MP × SP | 1, 18 | 3.45 | .079 | .161 |
| TT × SP | 1, 18 | .094 | .76 | .005 |
| MP × TT | 1, 18 | .72 | .41 | .039 |
| MP × TT × SP | 1, 18 | 11.44 | .003** | .389 |

MP = menstrual phase; TT = target type; SP = sex of prime.

**Table S3. Results of a mixed ANOVA with low attractive primes in Experiment 2.**

| Source | df | F | Sig. | η*_p_^2^* |
| --- | --- | --- | --- | --- |
| MP | 1, 18 | 1.39 | .25 | .072 |
| TT | 1, 18 | .84 | .37 | .044 |
| SP | 1, 18 | .012 | .91 | .001 |
| MP × SP | 1, 18 | .34 | .57 | .018 |
| TT × SP | 1, 18 | 2.41 | .14 | .118 |
| MP × TT | 1, 18 | 2.30 | .15 | .113 |
| MP × TT × SP | 1, 18 | .58 | .46 | .031 |

MP = menstrual phase; TT = target type; SP = sex of prime
